# Comparative and Optimized Chemical Synthesis of AgNPs for Improved Surface Reactivity and Potential Biosensing Applications

**DOI:** 10.3390/nano15231749

**Published:** 2025-11-21

**Authors:** Alexandra Nicolae-Maranciuc, Ioana Andreea Brezestean, Septimiu-Cassian Tripon, Andreea Campu

**Affiliations:** 1 Academy of Romanian Scientists, Ilfov No. 3, 050044 Bucharest, Romania; alexandra.nicolae@ulbsibiu.ro; 2 Institute for Interdisciplinary Studies and Research (ISCI), Lucian Blaga University of Sibiu, Victoriei Blvd. No. 10, 550024 Sibiu, Romania; 3 Research Center for Complex Physical Systems, Faculty of Sciences, Lucian Blaga University, Doctor Ion Rațiu No. 5-7, 550012 Sibiu, Romania; 4 Department of Molecular and Biomolecular Physics, National Institute for Research and Development of Isotopic and Molecular Technologies, Donat No. 67-103, 400293 Cluj-Napoca, Romania; ioana.brezestean@itim-cj.ro; 5 RDI Laboratory of Applied Raman Spectroscopy, RDI Institute of Applied Natural Sciences (IRDI-ANS), Babes-Bolyai University, Fântânele No. 42, 400293 Cluj-Napoca, Romania; 6 Electron Microscopy Center “Prof. C. Craciun”, Babes-Bolyai University, Clinicilor No. 5-7, 400006 Cluj-Napoca, Romania; septimiu.tripon@itim-cj.ro; 7 Electron Microscopy Integrated Laboratory, National Institute for Research and Development of Isotopic and Molecular Technologies, Donat No. 67-103, 400293 Cluj-Napoca, Romania

**Keywords:** silver nanoparticles, nanosize, chemical reduction, TSC, sodium borohydride, optimized synthesis, biosensing, surface reactivity, biomedical applications

## Abstract

Silver nanoparticles are metallic particles with very small dimensions and excellent optical, electrical and biological properties. Lately, they have shown promising results in biosensing applications. In the material’s fabrication, the synthesis parameters remain the main aspect to be considered once a certain application is targeted. Therefore, this work presents the synthesis of silver nanoparticles using a chemical reduction based on various volumes of reducing and stabilizing agents. The multiple synthesis methods proposed were tested and optimized in order to achieve the best results for further biosensing applications. In this regard, sodium borohydride (NaBH_4_) was used as reducing agent in volumes of 400 μL and 1 mL, while trisodium citrate (TSC) was proposed in much smaller volumes of 10, 20, and 50 μL. The optical and morphological analysis obtained from UV-VIS and TEM microscopy confirmed the formation of nanoparticles in case of all synthesis. The average diameters of silver nanoparticles were in the range between 21 and 27 nm, with high homogeneity for the samples with 20 and 50 μL of TSC. FT-IR analysis confirmed the TSC functionalization on the AgNPs’ surface. SERS analysis and the bulk sensitivity method also showed good surface results, leading to the assumption that both reducing and stabilizing agents can influence the final properties of the material. LSPR biosensing of para-aminothiophenol was tested, and was proven to have detection capabilities at concentrations as low as 10^−7^ M. Overall, the results proved that the synthesis method with a smaller amount of reducing agent and a moderate quantity of stabilizing agent has superior properties for biosensing applications.

## 1. Introduction

Nanomedicine, an innovative field which combines nanotechnology with medical sciences, has recently achieved powerful results in improving humans’ lives [1,2]. Offering a wide range of applications, nanomaterials are known for their small dimensions and improved properties. Their high surface-area to volume, enhanced biocompatibility, and versatility have led to an increase in the amount of research on various materials used in nanomedicine [3]. Nanoparticles are nanoscale structures with dimensions typically under 100 nm which possess high optical, electrical, or chemical activity [1,4]. Metallic nanoparticles, a complex category of nanomaterials used in biomedical applications, have shown promising results in healthcare, drug delivery, biosensing or tissue engineering applications [5].

Silver nanoparticles (AgNPs) are metallic inorganic nanoparticles with superior optical, electric and antibacterial properties [6]. In nanomedicine, AgNPs are well known for their ability to inhibit bacterial proliferation and to offer a strong antibacterial effect [7,8]. Since they are an interesting alternative to antibiotics, most of the literature studies that focus on them have investigated the conditions in which AgNPs exhibit this antimicrobial effect on Gram-positive or Gram-negative bacteria depending on the synthesis method performed [9,10,11]. In contrast, AgNPs have also shown great results in nanoplasmonic sensing and detection applications. In biosensors, AgNPs are mainly used due to their localized surface plasmon resonance (LSPR) property [12], which is caused by the free movements of electrons once the sample is excited with visible light [13]. Depending on the size, shape or surface, LSPR can change, leading to the identification of more in-depth information about the suspensions tested or their interactions with various other small molecules [14,15]. These main factors are strongly correlated with the synthesis method, so the controlling the synthesis parameters is essential to achieve the highest yield of sensing with respect to the employed detection technique. Therefore, based on possible shifts in plasmonic bands, biosensors fabricated with AgNPs can detect modifications in absorbance once the target is attached to their surface [13]. Nowadays, various possibilities have been exposed for AgNPs biosensors’ fabrication, depending on the detection technique. In biomedical applications, electrochemical [16] and optical [17] approaches showed the most promising results, offering specific detection at low concentration and improved biosensor performance [13]. For instance, Mandal et al. [18] developed an electrochemical biosensor by modifying a glassy carbon electrode with AgNPs for the early detection of prostate cancer. The results of the study proved the AgNPs’ nanosize dimensions and their proper surface, while a very small detection limit of 1 pg/mL compared to other existent biosensors for prostate cancer identification was assessed. Demonstrating an incredible sensitivity of about 0.38 µA/pg mL^−1^ and a detection of very low concentrations of antigen, the proposed biosensor proved to be a cost-effective intelligent tool for cancer therapy [18]. Jia et al. [19] proposed a biosensor based on a AgNP embedded polymer–zirconium-based metal–organic framework (polyUiO-66@AgNPs) for the detection of respiratory virus SARS-CoV2. The AgNPs were prepared through a chemical hydrothermal method, while the ligand agent in the biosensor’s fabrication was a small molecule of carboxylic acid. Based on the high porosity and improved surface properties of AgNPs, the integration of biological molecules such as aptamers and antibodies was achieved with high precision. The system proved to have an extremely low detection limit of 23.4 fg mL^−1^, with good stability and excellent reproducibility [19].

The main aspect to consider in biosensing and biological applications is the synthesis method chosen for AgNPs, since their properties are strongly correlated with the fabrication techniques and the parameters used. Generally, AgNPs can be obtained through physical, chemical, or biological synthesis, depending on the reagents used [20,21]. Chemical routes are most frequently used in the literature because they can be easily obtained in the laboratory, and they are cost-effective and reproductible [22]. Between the chemical strategies, chemical reduction is often preferred, since the reaction time is low, and the method can be easily adjusted. In chemical reduction, a metal salt is introduced in the reaction as a precursor, a reducing agent is used to reduce the metal ions, and in the final step, a stabilizing agent is added to increase the stability of the particles obtained [23]. Over time, various reagents were proposed depending on the application that was targeted. For example, Khatoon et al. [24] mediated the synthesis of AgNPs using NaBH_4_ as both reducing and stabilizing agents. The physical–chemical characterization proved able to obtain nanosized AgNPs in a range of 10 nm–50 nm with spherical shape and crystalline structure [24]. In a recent study, Sreelekha et al. [25] proposed a comparison between chemical reduction using trisodium citrate and green chemistry. The chemical synthesis performed in the article showed dimensions between 9 and 14 nm compared to the green synthesis, for which higher diameters were identified. AgNPs exhibited enhanced efficiency in antioxidant activity for both synthesis methods, confirming their positive effect in biomedical applications [25]. The choice of the proper parameters is extremely important, since AgNPs suspensions are sensitive to the concentration of solution adjusted. Also, the reagents used can influence the final surface of AgNPs, leading to inappropriate detections in case of biosensors or an insufficient effect in case of antibacterial applications.

In most of the literature studies, the final purpose of the synthesized AgNPs is for antimicrobial applications. Their implementation in sensing applications is underinvestigated compared to other type of nanoparticles; hence, less studies on surface chemistry have been reported for AgNPs. The size and the shape are extremely important, since they can affect the final properties of the material. The optical properties, the plasmonic features, and even the toxicity level depend on the synthesis process. Lately, several authors have described the chemical AgNPs synthesis and their impact in stabilizing AgNP-based colloidal suspensions [26,27,28]. However, most of the existing studies rely on the investigation of multiple chemical reagents in similar concentrations or the investigation of the influence of one specific agent tested. There are a few studies in which both the reducing agents and the stabilizing agents are investigated in such variations in concentration in order to find the best condition to obtain nanosized AgNPs. Furthermore, the efficacy of AgNPs in biosensing applications is strongly dependent on their ability to efficiently graft target molecules onto their surface; thus, their surface chemistry is of the utmost importance in the development of versatile and sensitive biosensors. Nanoparticles’ surface reactivity is influenced by the synthesis agents and their interaction; hence, an in-depth investigation of the synthesis parameters and their effect on the AgNPs’ surface chemistry is required to establish the biosensor fabrication approach and corresponding detection analytical tool, as even a small amount of reagent can change the final properties of the particles and, implicitly, their sensing abilities. Moreover, most studies rely on biosensors’ fabrication using gold nanoparticles, which have been studied for many years now. There are fewer publications describing the implementation of AgNPs in sensing applications and surface chemistry; therefore, this work could offer promising results and in-depth information for future fabrication of biosensors using AgNPs.

To respond to the above-mentioned requirements, the aim of this paper is to compare and to optimize the chemical reduction of AgNPs for further potential (bio)sensing applications. In this regard, various ratios of reducing (NaBH_4_) and stabilizing (TSC) agents were proposed to identify their impact on nanoparticles’ shape, dimension, and sensitivity. Through this approach, we aim to achieve a balance between performance and nanosize dimensions, leading to an improvement in the surface reactivity of the AgNPs suspensions. To obtain as much information as possible, two different volumes of 400 μL and 1 mL of NaBH_4_ were tested, while for TSC, three variations of 10, 20, and 50 μL were proposed. The influence of both reagents was determined from an AgNPs dimensional perspective, while the sensitivity properties were assessed for all synthesis variations. Surface chemistry studies were performed to evaluate the AgNPs’ capabilities to efficiently graft target molecules onto their surface and, implicitly, to assess their ability to be implemented as signal transducers for different detection techniques in order to achieve the best conditions for further potential biosensing applications.

## 2. Materials and Methods

### 2.1. Materials

The materials involved in this study were purchased from Sigma-Aldrich (Germany), according to Table 1. The materials were used without any further purification and the solvent used was ultrapure water. The para-aminothiophenol (p-ATP) used for the biosensing test was purchased from Sigma-Aldrich (Germany), as well.

The main goal of this work is the identification of the best conditions for further sensing applications. Therefore, various ratios were tested for the chemical synthesis in order to optimize the final synthesis. All the chemical reductions proposed in this study were achieved by maintaining the same AgNO_3_ concentration. In addition, both the reducing and stabilizing agents’ volumes were alternated, as it described in Table 2.

### 2.2. AgNPs Chemical Syntheses

**Figure 1:**
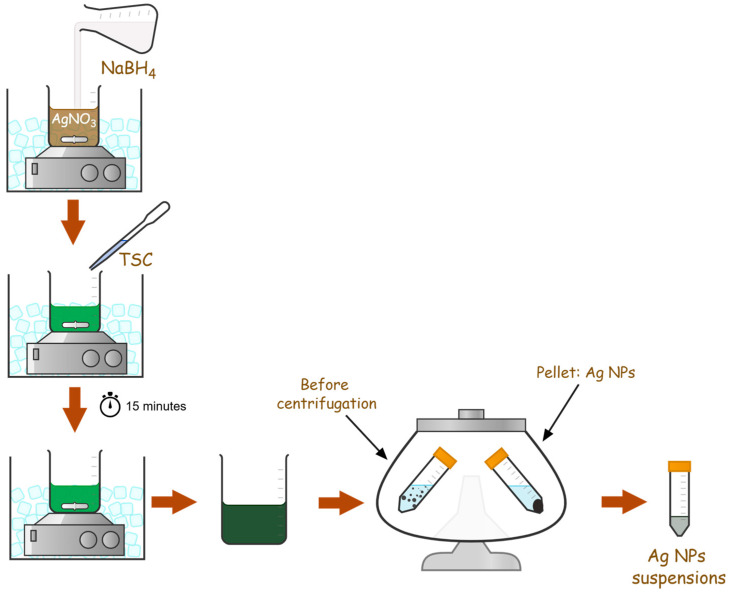
Schematic illustration regarding AgNPs chemical synthesis.

For this study, we performed six chemical reductions using different ratios of reducing and stabilizing agents, as described in Table 2. The protocol of the reactions is described in Figure 1. All the syntheses were performed in a cold environment using ice to maintain the low temperature required when using NaBH_4_.

A 3 mM AgNO_3_ solution was first obtained by using ultrapure water as solvent. Then, 1% TSC and 30 mM NaBH_4_ were freshly prepared by using cold ultrapure water as solvent. Each synthesis was performed in a 20 mL Berzelius glass, which was kept on ice in the magnetic stirrer. For each sample, 400 μL and 1000 μL of NaBH_4_, respectively, were added drop by drop into the AgNO_3_ solution. Immediately after the color changes, TSC 1% was added in quantities of 10, 20, or 50 μL and each solution was allowed to stir for 15 min. The mixing was stopped after 15 min once the solution had achieved a green color. Further, a 2:50 dilution of each synthesis was performed in ultrapure water, and the samples were centrifuged at 4000 rpm (2571× *g*) for 40 min. Each pellet was resuspended in 5 mL of pure water and was kept at 4 °C for further analysis.

### 2.3. Determination of the Bulk Sensitivity

To assess the sensing capabilities of the AgNPs, their *bulk* sensitivity was evaluated. In this context, the AgNPs were centrifuged for 40 min at 4000 rpm and the supernatant was removed, while the AgNPs pellet was redispersed in water–glycerol mixtures containing different glycerol concentrations, i.e., 0, 20, 40, 60, and 80% glycerol, in order to change the refractive index (RI) in the close vicinity of the AgNPs. By exposing the AgNPs to the water–glycerol mixtures, the RI varies from 1.333, corresponding to the RI of water, to 1.44, as analytically determined according to the Lorentz–Lorentz equation [29]. After redispersion, LSPR spectra were recorded. The recorded shift in the LSPR band was plotted against the RI, followed by the execution of a linear regression analysis, the slope of which indicates the *bulk* refractive index sensitivity of the studied AgNPs. The determination of their *bulk* sensitivity was realized in triplicate.

### 2.4. Biosensing Protocol

To test the AgNPs’ capabilities for potential (bio)sensing applications, samples A2 and A5 were functionalized with para-aminothiophenol (p-ATP), a well-known small molecule which binds to the metallic surface through the thiolated active group. The AgNPs were purified as described above and exposed to various ethanolic p-ATP concentrations, specifically 10^−4^, 10^−5^ 10^−6^, and 10^−7^ M. Following the functionalization, UV-VIS-NIR spectra were recorded and the LSPR red-shifts were extracted and plotted against the p-ATP concentration. These tests were realized in triplicate.

### 2.5. Characterization Techniques

#### 2.5.1. UV-VIS Spectroscopy

For the investigation of all AgNPs synthesized in this work, a Specord 210 Plus UV–VIS spectrophotometer provided by Analytik Jena (Jena, Germany) was used. The suspensions of AgNPs were introduced into a quartz cuvette, along with a water sample serving as a reference. The absorption was carried out in the range of 300 nm to 900 nm.

#### 2.5.2. TEM Morphological Characterization

The morphology of the AgNPs in aqueous solution was investigated using a Hitachi HD-2700 scanning (Tokyo, Japan) and transmission electron microscope, Aztec, Oxford Instruments (Abingdon, United Kingdom), which was able to reach a magnification of up to 10 million times in ultrahigh-resolution mode and a 200 kV electron acceleration. Prior to the TEM analysis, the samples were suspended in ethyl alcohol, deposited on a 3 mm diameter electrolytic carbon-coated grid with a mesh of 300 using an automatic pipette, and left at room temperature for the supernatant to evaporate, leaving the AgNPs immobilized onto the grid’s carbon film. To decontaminate the samples, a Hitachi ZONE TEM ozone sample decontamination unit was employed. Further, at a set 200 kV accelerating voltage, microscopic TEM images at different magnifications were recorded. To determine the size of the AgNPs, the TEM images underwent an analysis using the commercially available ImageJ (version 1.46r) toolkit. Based on the extracted diameters of 100 to 200 AgNPs, particle size distribution histograms were realized.

#### 2.5.3. SERS Spectroscopy

Raman spectra were collected using an i-BWTEK Raman spectrometer (Newark, Delaware, USA) equipped with a liquid sample holder (working distance = 20 mm). Samples were placed in 1 mL clear glass vials (40 × 8.2 mm; Llg Labware, Meckenheim, Germany). A 532 nm laser excitation was used, and spectra were acquired over the 4000–100 cm^−1^ range. Each spectrum was obtained with an integration time of 10 s and consisted of a single acquisition without signal averaging. The laser power on the sample was maintained at 10% (3 mW) of the instrument’s maximum output (30 mW) to minimize local heating or photodegradation effects.

#### 2.5.4. FT-IR Spectroscopy

FT-IR spectroscopy was performed to detect the possible chemical modifications that appear in the synthesis process of AgNPs, while reducing and stabilizing agents’ influence was addressed. This characterization was performed using an FT-IR ALPHA spectrophotometer with an ATR crystal (Bruker, Billerica, MA, USA). The analysis was carried out on the powdered samples, which were previously dried in the oven. The analysis was based on 24 scan times at a resolution of 4 cm^−1^ with a wavelength in the range of 400–4000 cm^−1^, while the absorbance of each sample from A1 to A6 was recorded.

## 3. Results and Discussions

### 3.1. Optical and Morphological Characterization: UV-VIS Analysis and TEM Microscopy

**Figure 2:**
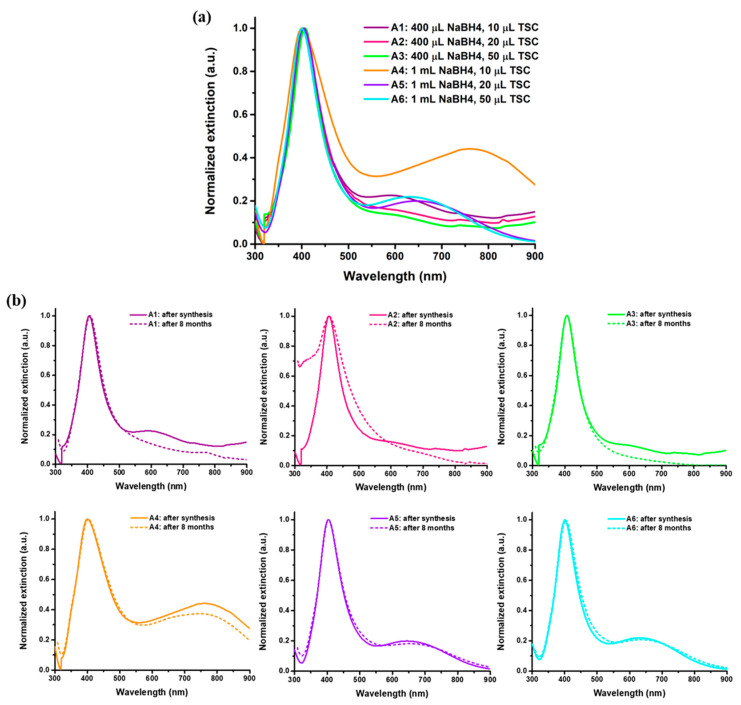
(**a**) LSPR spectra of the as-synthesized AgNPs in aqueous solution. (**b**) Normalized extinction spectra after synthesis compared to the extinction spectra recorded after 8 months from the growth of the nanoparticles, proving the stability of all colloidal AgNPs samples.

**Figure A1:**
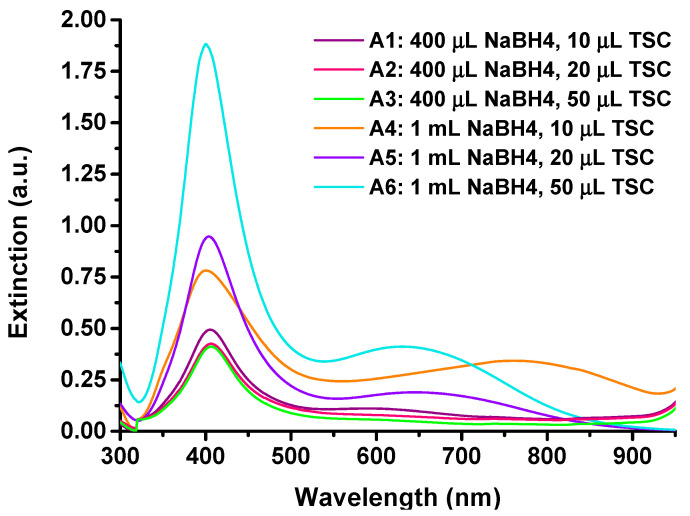
Raw extinction spectra after synthesizing all colloidal AgNPs samples.

Firstly, the as-synthesized AgNPs in aqueous solution were thoroughly optically and morphologically characterized. UV-VIS absorptions of AgNPs synthesized using various ratios of reducing and stabilizing agents are shown in Figure 2a. The raw extinction spectra of all colloidal AgNPs samples are found in Appendix A, Figure A1.

The LSPR spectra exhibit two extinction bands. Concretely, the formation of AgNPs is confirmed by the well-defined first extinction band for all six samples. The high intensity of the main peak recorded in the range of 400 nm to 405 nm suggests the formation of nanosized AgNPs, confirming that, for all tested variations in the reducing and stabilizing agents, the syntheses were successfully realized. The identification of the AgNPs characteristic LSPR peak sustain that both TSC and NaBH_4_ used in the reactions create a proper environment for the reduction of the Ag ions and further stabilization of AgNPs. The second LSPR band exhibited by the synthesized AgNPs can be assigned to larger nanostructures, as the LSPR is highly dependent on the size of the nanoparticles. According to the Mie theory, an increase in size can be observed as a translation of the LSPR to higher wavelengths [30]. The presence of both LSPR bands indicates the presence of both smaller and larger nanoparticles with respect to the differences between the reducing and stabilizing agents’ volumes. Regarding the AgNPs shape, LSPR bands suggest the formation of predominantly spherical particles due to the main peak observed around 405 nm. The second shoulder observed at higher wavelengths suggests the formation of other shapes and morphologies [31]; however, its small intensity exposes the theory in which only small parts of the particles are found in different shapes. Furthermore, the second LSPR peak is identified mostly for samples A4–A6, leading to the assumption that an increase in the reducing agent can lead to a variation in AgNPs’ shape. The results are confirmed by TEM images, in which slightly larger nanoparticles are also observed. For the samples using a higher amount of reducing agent (NaBH4), a higher polydispersity is suggested. Samples A1–A3 present a smoother extinction shoulder, while for samples A4–A6 this shoulder is more defined and shifted at even higher wavelengths. These results are in good correlation with literature studies where a range of 400–450 nm is well known to be specific for AgNPs synthesis [32,33].

To further characterize the AgNPs colloidal solutions, the full-width at half maximum (FWHM) and the AgNPs molar concentration were determined (Table 3). For the calculation of the concentration, for samples A1, A2, A3, and A5, the extinction coefficient was 1.45 × 10^10^ L/mol·cm, while, for samples A4 and A6, the value for the extinction coefficient was 4.18 × 10^9^ L/mol·cm, according to literature reports [34]. The FWHM values range between 61 and 100 nm, confirming the narrow LSPR response and, implicitly, the presence of prevalently individual AgNPs with similar sizes. Thus, the homogeneity in terms of size is confirmed. The molar concentrations determinations show that the use of higher amounts of reducing agent leads to a higher AgNPs synthesis yield.

Furthermore, the stability of the AgNPs over time was evaluated by recording their extinction spectra after 8 months of storage at 4 °C in the dark. Figure 2b shows a comparison between the extinction spectrum of the AgNPs after synthesis and after 8 months of storage. No significant modifications in the LSPR response are identified—the extinction spectra recorded after 8 months overlap with the ones acquired after synthesis, thus proving the high stability of the colloidal AgNPs solutions in time and, implicitly, their long shelf-life.

**Figure 3:**
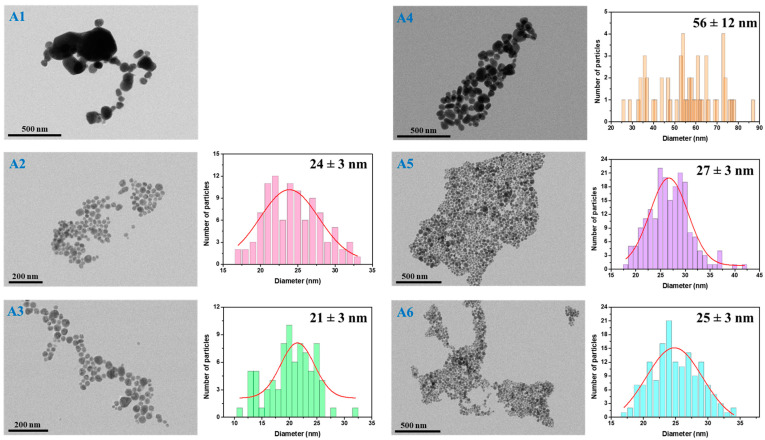
Representative TEM microscopic images and their corresponding particle size distribution histograms for all synthesized colloidal AgNPs in aqueous solution.

**Figure A2:**
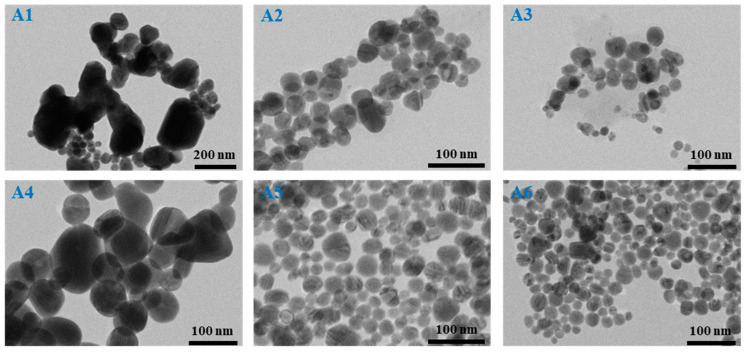
Representative TEM microscopic images at higher magnification for all AgNPs colloidal samples.

The next step was to evaluate the as-synthesized AgNPs in terms of their morphology and size; thus, TEM microscopy was employed for all samples. Microscopic TEM images were recorded and analyzed using the commercially available ImageJ toolkit. For the determination of the average AgNPs diameter, sets of 100 to 200 nanostructures were measured and represented as particle size distribution histograms. Figure 3 shows representative TEM microscopic images of all AgNPs with their corresponding size distribution histograms. Inserted into the histograms are the determined average diameter sizes with the standard deviations. Additional TEM microscopic images at higher magnification are found in the Appendix A, Figure A2.

As indicated by UV-VIS observations, all samples exhibit the formation of AgNPs with differences according to the reducing–stabilizing agents’ ratio, with the TEM analysis being in good agreement with the optical determinations. Specifically, the proposed synthesis strategies lead to the formation of a rather high yield of spherical nanostructures and neglectable amounts of byproducts, proving to have high homogeneity in terms of shape.

Moreover, samples A5–A6, which were obtained with a higher amount of reducing agent, show slightly larger nanoparticles than their counterparts (A2–A3), where less NaBH_4_ was added. However, the polydispersity in terms of size is lower in the case of the A2–A3 samples. Additionally, a higher amount of TSC stabilizing agent induces a decrease in the AgNPs’ diameter for both NaBH_4_ concentrations. In the case of A1 and A4 samples, the polydispersity is highest, showing the formation of both small and considerably larger nanostructures, making the realization of the particle size distribution histograms rather difficult. For the synthesis of these samples, 10 μL of TSC was used, leading to the assumption that the stabilizing agent concentration was too low and, therefore, the AgNPs tended to aggregate and form larger nanostructures. The reducing agent concentration also plays an important role, as a higher NaBH_4_ and lower TSC concentration induce growth of up to around 90 nm AgNPs with an average diameter of 56 ± 12 nm. The reduction reaction in the presence of both lower NaBH_4_ and TSC concentrations produces a higher number of Ag nanostructures with diameters over 100 nm.

Furthermore, samples A2–A3 and A5–A6 exhibit similar LSPR band positions located at around 400–405 nm, suggesting the presence of nanosized nanoparticles with comparable diameters. These determinations are supported and confirmed by the particle size distribution histograms, as the diameter size varies between 27 and 21 nm with 3 nm standard deviations. A 30 μL increase in the TSC amount slightly decreases their size; however, the increase in the reducing agent leads to a higher yield of AgNPs. Therefore, according to TEM microscopy, synthesis A2 and A5, in which 20 μL of TSC were used, seems to be the most promising choice for further applications.

### 3.2. FT-IR Analysis

**Figure 4:**
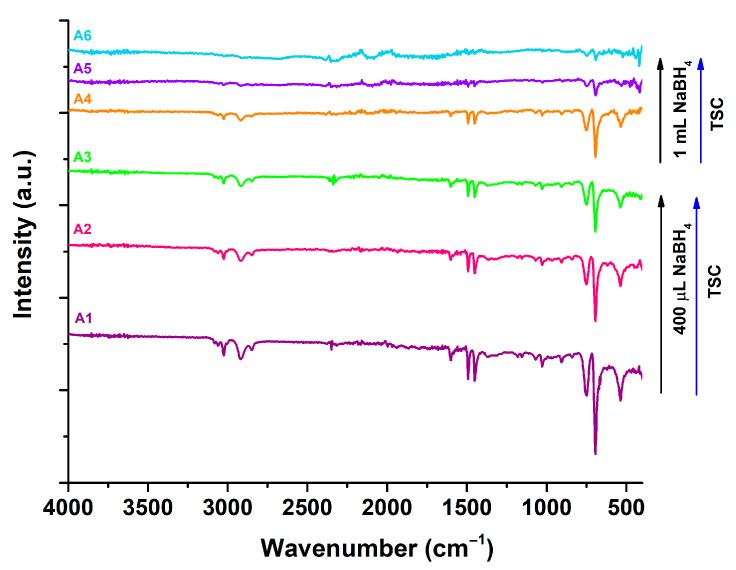
FTIR spectra of synthesized colloidal AgNPs in aqueous solution.

FT-IR analysis was performed for all six syntheses in order to identify the main chemical groups found in AgNPs and to observe the influence of the reducing and stabilizing agents on the variations in the compounds found on their surfaces and inside the particles’ suspensions. The results of FT-IR spectroscopy, observed in Figure 4, suggest a real difference between samples A1–A3 and A4–A6.

FT-IR spectroscopy reveals the vibrational bands for various chemical molecules involved in the reducing and stabilizing process. The peak identified at 3120 cm^−1^ is associated with the vibrational O-H groups from the citrate, while the peaks from 2910 and 2855 cm^−1^ are correlated with C-H stretching from the chemicals used in the reaction [35]. The functionalization with TSC for all synthesized AgNPs is confirmed by the peak recorded at 1680 cm^−1^ and 1580 cm^−1^, which is associated with C=O and C-C stretching, indicating the presence of a -COO- chemical group on the surface of each nanoparticle [36]. Samples A1–A3 presents higher intensities for these peaks compared to their counterparts A4–A6; therefore, a bigger quantity of TSC could be found on their surface. The last peaks identified around 755 and 690 cm^−1^ suggest the presence of C=C bending or a symmetric stretching band of B-O [37].

From the results obtained, it is suggested that NaBH_4_ influences the final surface properties of the nanoparticles. Samples A1–A3, which contain less reducing agent, show a larger quantity of more intense bands compared to samples A4–A6, in which the intensity of the peaks is decreasing. Furthermore, TSC’s influence is also confirmed, since the samples with more stabilizing agent show less absorbance in IR. These results could be explained by a possible mechanism between the two reagents used. Adding a higher quantity of NaBH_4_ to the reaction can lead to a faster reduction. Therefore, a higher number of synthesized particles can lead to a larger surface which could be further covered by TSC. Since the TSC is added in very small amounts, each particle will receive less TSC on their surface, leading therefore to smaller intensities in FT-IR spectra for the samples where NaBH_4_ was added in larger quantities. These assumptions are sustained by the differences between syntheses A1 and A3, for example, where the same amount of TSC was added. The sample with more NaBH_4_ shows stronger absorption in IR. Also, it could be the intercalation of TSC between two or more particles when more NaBH_4_ is used, leading to a decrease in the vibrations of the molecules. This would lead to less identification of TSC on the surface using FT-IR spectroscopy, since it will also be trapped between the particles. However, for this hypothesis to be sustained, higher intensities in further SERS analysis should be identified. The SERS results proved that a bigger amount of reducing agent will lead to higher densities of TSC on AgNPs surfaces; therefore, this mechanism is highly likely to happen.

### 3.3. SERS Analysis of the AgNPs Surface

In order to assess the AgNPs’ capabilities to efficiently detect modifications in their close vicinity and, thus, operate as signal transducers in biosensing applications, their surface was characterized using SERS spectroscopy. Concretely, the next step was to record the SERS response of the as-synthesized AgNPs using a Raman spectrometer. For this purpose, the colloidal AgNPs in aqueous solutions were exposed to a laser with an excitation wavelength of 532 nm with a set power at 10% and an integration time of 10 s.

**Figure 5:**
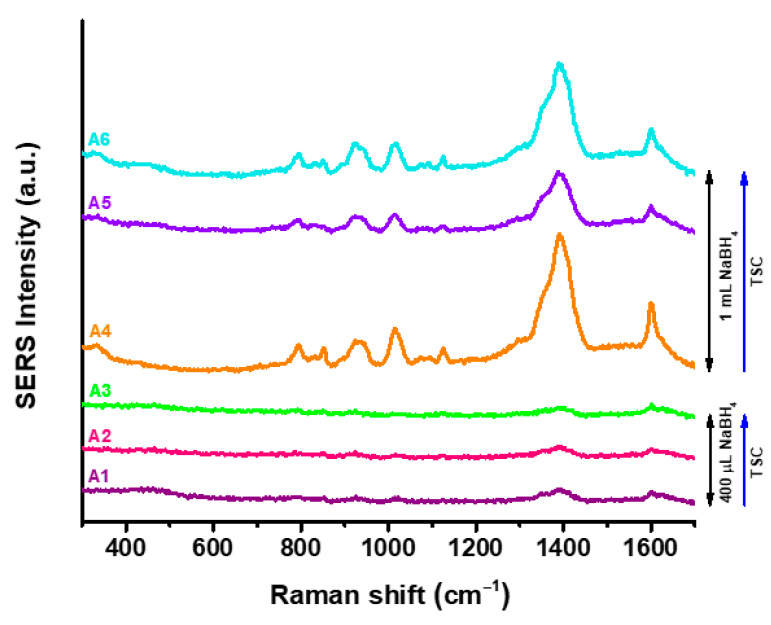
SERS spectra of synthesized colloidal AgNPs in aqueous solution.

From the SERS spectra recorded for each of the synthesized AgNPs (Figure 5), it can be observed that AgNPs obtained using 400 µL of NaBH_4_ have a weak SERS response irrespective to the TSC concentration, in agreement with the literature [38]. For a higher concentration of reducing agent, characteristic TSC bands appear in the SERS spectra, indicating the presence of a large amount of stabilizing agent on the nanoparticles’ surface. Specifically, in the SERS spectra of A4–A6, the following characteristic vibrational modes can be identified: the bands located at 830–852 cm^−1^ are assigned to C-C stretching vibrations; those at 925–943 cm^−1^ are indicative of C-COO stretching modes; at 1125 cm^−1^, stretching vibrations of C-OH are identified; the band at 1391 cm^−1^ corresponds to symmetrical stretching vibrations of COO; while the COO asymmetric vibrational modes are indicated by the Raman band located at 1600 cm^−1^ [39]. Thus, the presence of TSC on the surface of the AgNPs is specifically identified and confirmed. However, the reducing agent concentration influences the coverage of the metallic surface with TSC, as the characteristic Raman bands of the stabilizing agent are considerably enhanced when higher amounts of NaBH_4_ are used to reduce the silver ions. Thus, for SERS biosensing applications, the AgNPs with lower amounts of NaBH_4_ are better candidates of choice, as the TSC signal does not overlap with or cover the Raman vibrational bands of the target analytes.

The differences between FT-IR and SERS appear due to the basic principles of the technique. In FT-IR, the total amount of compounds is recorded, yet for SERS only the surface of the particle is analyzed. Therefore, in the case of samples A4–A6, where the peak intensity is lower in FT-IR, but higher in SERS, TSC is most likely to be distributed between two or more particles, not only on their surface. FT-IR spectroscopy and SERS analysis correlation confirmed the functionalization and stabilization with TSC on AgNPs surface, a process which could be the main reason for the optimal stability of all AgNPs samples. For further analysis, the samples A2 and A5 were chosen in order to evaluate the optical sensing capabilities of both AgNPs categories.

### 3.4. Study of the AgNPs Bulk Refractive Index Sensitivity (RIS)

**Figure 6:**
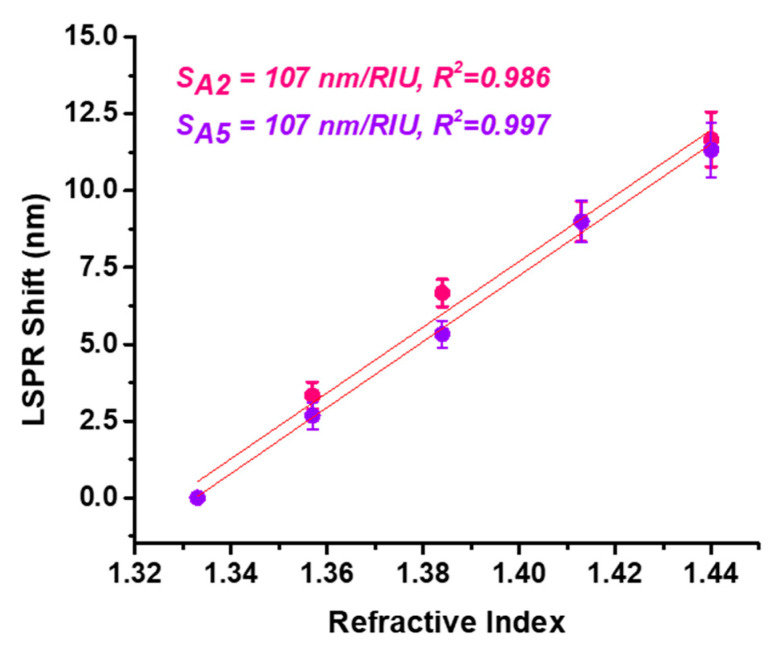
Graphical representation of the LSPR red-shift with respect to the refractive index of the medium in the close vicinity of the nanoparticles, along with their corresponding linear regressions.

Lastly, the *bulk* refractive index sensitivity (RIS) of samples A2 and A5 was studied to determine their optical biosensing capabilities. Specifically, the sensitivity of the nanoparticles to changes in the microenvironment in the nanoparticles’ close vicinity was evaluated. For this aim, the dielectric medium of the nanoparticles previously in aqueous solution was changed with water–glycerol mixtures of different concentrations, i.e., 0, 20, 40, 60 and 80% glycerol. The LSPR property of metallic nanoparticles is dependent on the refractive index of the medium in which the nanoparticles are located; thus, an increase in the refractive index leads to a red-shift in the LSPR band. By employing different water–glycerol mixtures, the refractive index was changed from 1.333 corresponding to water to 1.44 corresponding to an 80% glycerol concentration. The LSPR response was monitored for each glycerol concentration, followed by the extraction of their spectral positions. With an increasing refractive index, the LSPR of the A2 sample red-shifted up to 8 nm for the 80% water–glycerol mixture, whereas for A5 the extinction band was translated to higher wavelengths with 12 nm, exhibiting higher sensitivity to refractive index changes in the microenvironment in the AgNPs’ proximity. Further, the LSPR red-shift with respect to 0% glycerol concentration was plotted as a function of the refractive index (Figure 6). From the obtained linear regressions, the RIS values for AgNPs were extracted as the slope of the fitted lines resulting in RIS of 107 nm/RIU for both samples. The R^2^ coefficients show the good correlation of the fit. These results prove the strong influence of the synthesis parameters and the need to optimize the methods applied in order to achieve the best results for further biosensing applications.

### 3.5. “Proof-of-Concept” Biosensing

**Figure 7:**
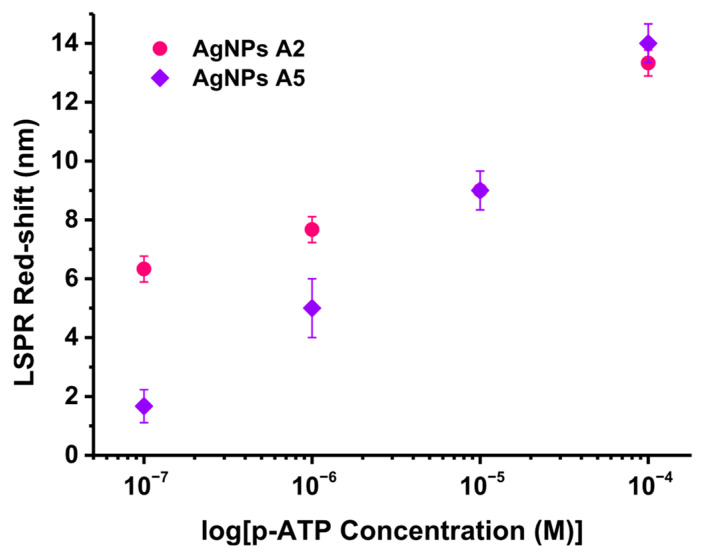
Graphical representation of the LSPR red-shift with respect to the logarithm of the tested p-ATP concentrations along with their corresponding linear regressions.

Finally, the biosensing capabilities of the A2 and A5 AgNPs samples were tested. p-ATP, a small well-known molecule, was chosen to be grafted onto the AgNPs due to its thiolated active functional group which binds to the metallic surface. Concretely, the AgNPs were exposed to various ethanolic p-ATP concentrations from 10^−4^ to 10^−7^ M, followed by the recording of their extinction spectra. The biosensing test was realized in triplicate. From the comparison to the LSPR responses of the unfunctionalized AgNPs, the LSPR red-shifts were extracted and graphically represented with respect to the p-ATP tested concentrations (Figure 7). The functionalization with p-ATP induced red-shifts from 13 nm for the highest concentration to 6 nm for the lowest concentration in the case of AgNPs sample A2, while for sample A5 the red-shifts varied from 14 nm (highest concentration) to 2 nm (lowest concentration). Both samples showed LSPR biosensing capabilities; however, sample A2 shows better performances compared to A5, indicating that concentrations below 10^−7^ M can be achieved.

Thus, the results presented in this work have proved that all six syntheses can be considered optimal for AgNPs fabrication. The UV-VIS spectra have shown specific LSPR peaks associated with nanometric dimensions and predominantly spherical shape, while the syntheses performance was confirmed by the high stability of the AgNPs in time (up to 8 months). The variation of TSC and NaBH_4_ suggested a real impact on the AgNPs surface, leading to the assumption that their size and shape can be influenced by the conditions in which the synthesis is performed. Even if all syntheses proved good results, samples A2 and A5, the ones with a moderate amount of TSC added (20 μL), exhibit better performances for further sensing applications. NaBH_4_ contribution has also been established for detection applications, as we demonstrated that sample A2, the one with less reducing agent (400 μL) and moderate stabilizing agent (20 μL), exhibits the best performance for biosensing applications.

## 4. Conclusions

In summary, this work focused on the comparison and optimization of the chemical synthesis approach used for AgNPs growth in order to assure their implementation in biosensing applications. Therefore, colloidal AgNPs in aqueous solution were obtained by introducing different reducing and stabilizing agent concentrations in the Ag ions reduction reaction. All proposed synthesis parameters lead to the formation of AgNPs confirmed by both optical and morphological analysis. TEM microscopy has established average diameters of 27 and 21 nm with 3 nm standard deviations and high homogeneity in terms of shape for the samples with 20 and 50 μL of stabilizing agent. FT-IR analysis showed a correlation between NaBH_4_ and TSC leading to the assumption that all samples present TSC at their surface, but in different amounts. The analysis of the surface chemistry using SERS has shown that a higher amount of reducing agent promotes a higher density of stabilizing agent at their surface; thus, for SERS biosensing, the AgNPs obtained with a lower amount of reducing agent are more appropriate. Finally, a RIS of 78 nm/RIU for sample A2 and 107 nm/RIU for sample A5, respectively, were determined establishing the superiority for optical biosensing of the AgNPs synthesized with higher concentrations of reducing agent. Lastly, the LSPR biosensing performances were tested, showing the ability to detect target analytes below 10^−7^ M and exhibiting a better performance for sample A2, the one with a smaller amount of NaBH_4_ and a moderate amount of TSC. The results provide a better understanding of the required optimization in concordance with the desired biosensing application and, more importantly, the detection technique to be implemented to achieve high sensitivity and accurate biosensing.

## Figures and Tables

**Table 1:** Materials involved in AgNPs syntheses.

Chemical Reagent	Chemical Formula	Purity	Form
**Silver nitrate**	AgNO_3_	99% ACS	powder
**Trisodium citrate dihydrate (TSC)**	C_6_H_5_Na_3_O_7_ × 2H_2_O	99%	powder
**Sodium Borohydride**	NaBH_4_	97%	liquid

**Table 2:** Description of the samples and the chemical composition of AgNPs colloidal solutions.

Sample	AgNO_3_ 3 mM (mL)	NaBH_4_ 30 mM (μL)	TSC 1% (μL)
**A1**	20	400	10
**A2**	20	400	20
**A3**	20	400	50
**A4**	20	1000	10
**A5**	20	1000	20
**A6**	20	1000	50

**Table 3:** The LSPR band, full-width at half-maximum (FWHM) and nanoparticle concentration for all synthesized samples.

Sample	LSPR (nm)	FWHM (nm)	AgNPs Concentration (M)
**A1**	405	61.47	0.34 × 10^−10^
**A2**	405	85.42	0.30 × 10^−10^
**A3**	405	87.23	0.29 × 10^−10^
**A4**	400	99.68	0.19 × 10^−9^
**A5**	404	78.88	0.66 × 10^−10^
**A6**	400	73.7	0.45 × 10^−9^

## Data Availability

Data are contained within the article.
